# Crystal structure of acylphosphatase from hyperthermophilic archaeon *Pyrococcus horikoshii* OT3

**Published:** 2004-09-01

**Authors:** Ken-ichi Miyazono, Yoriko Sawano, Masaru Tanokura

**Affiliations:** Department of Applied Biological Chemistry, Graduate School of Agricultural and Life Sciences, The University of Tokyo, 1-1-1 Yayoi, Bunkyo-ku, Tokyo 113-8657

**Keywords:** Acylphosphatase, archaea, crystal structure determination

## Abstract

Analysis of the hyperthermophilic archaeon *Pyrococcus horikoshii* OT3 genome database led to the discovery and cloning of acylphosphatase (ORF PH0305a). To elucidate the first structure of archaeal acylphosphatase, we determined the crystal structure of *P. horikoshii* acylphosphatase at 1.72 Å resolution. The space group of the crystals was *P*3_2_21, with unit-cell parameters *a* = *b* = 86.6 Å and *c* = 75.4 Å. The overall fold of *P. horikoshii* acylphosphatase was very similar to the structures of the eukaryotic enzymes. The conformation of putative active site was highly conserved.

## Introduction

Acylphosphatase (AcP; EC 3.6.1.7) is one of the smallest enzymes, with molecular weight of approximately 10000. AcP catalyzes the hydrolysis of acylphosphates. AcP has been implicated in the control of the glycolytic pathway, in pyrimidine biosynthesis, and in ion-pump activity.1)–3) This enzyme is widespread in all vertebrate tissues, in the form of two highly homologous isoenzymes: muscle type (MT) and organ common type (CT).4) MT-AcP is found mainly in the skeletal muscle and heart, whereas CT-AcP is found in all tissues such as erythrocyte, brain, and testis. These enzymes share more than 50% amino acid sequence identity. AcPs from non-vertebrate sources are also found in *Drosophila melanogaster* (AcPDro, AcPDro2)5),6) with about 40% sequence identity to human MT and CT AcPs.

The three-dimensional structures of horse MT-AcP and bovine CT-AcP have been determined by NMR and X-ray crystallography, respectively.7),8) Recently the crystal structure of *Drosophila melanogaster* AcP (AcPDro2) was determined.9) Regardless of the sources of AcPs, their structures are similar to one another, consisting of one very compact globular ***α***/***β*** fold with two ***α***-helices and a five-stranded ***β***-sheet. Site-directed mutation analysis and three-dimensional structural data revealed that two conserved residues, Arg23 and Asn41, are important for enzyme activity. Arg23 is involved in the binding of the phosphate moiety of the substrate, and Asn41 has been recognized as the catalytic residue involved in the orientation and stabilization of catalytic water molecule.8)

*Pyrococcus horikoshii* OT3 is a hyperthermophilic archaeon that was isolated from a hydrothermal fluid. *P. horikoshii* OT3 genome data indicate that this hyperthermophilic archaeon has the AcP gene (ORF PH0305a).10) The protein encoded by this ORF consists of 91 amino acid residues with a molecular weight of 10260.

To elucidate the first structure of archaeal AcP, we crystallized *P. horikoshii* AcP and determined its three dimensional structure by X-ray crystallography. The overall fold of *P. horikoshii* AcP was very similar to the structures of eukaryotic enzymes, except for the loop structure near the C-terminus. The structure of putative active site was highly conserved.

## Materials and methods

*P. horikoshii* AcP was overexpressed in *E. coli*, purified, and crystallized as described.11) Protein samples were concentrated to 10 mg/ml for crystallization. All crystallization experiments were performed using the sitting-drop vapor-diffusion method at 293 K, and 1 μl of protein solution was mixed with 1 μl of reservoir solution. The best crystals were obtained after 2 days using the following reservoir composition: 0.7–0.9 M K/Na tartrate and 100 mM citrate buffer (pH 5.5).

Crystals were transferred into a cryo-protectant solution containing 0.8 M K/Na tartrate, 100 mM citrate buffer (pH 5.5), and 20% ethylene glycol before being picked up and flash-cooled in a nitrogen stream. Diffraction data were collected at BL41XU in SPring-8 at 100 K using a MAR CCD detector system to a resolution of 1.72 Å. Data were processed with *DENZO*/*SCALEPACK*.12) The crystals belonged to hexagonal space group *P*3_1_21 or *P*3_2_21 with unit cell parameters *a* = *b* = 86.6 Å and *c* = 75.4 Å. Consideration of the values of *V*_M_ suggests that these crystals may have 2, 3, 4, or 5 molecules per asymmetric unit (*V*_M_ = 4.0, 2.7, 2.0, and 1.6 Å^3^ Da^−1^, respectively). 13)

The structure of the *P. horikoshii* AcP was determined by the molecular replacement method. Molecular replacement was performed with the program *MOLREP* from the CCP4 suites14) using the coordinates of bovine CT-AcP (PDB code 2ACY8)). *MOLREP* was run using the data with a resolution range of 30-3 Å in both space groups *P*3_1_21 and *P*3_2_21. The best solution was obtained when we searched two monomers in the asymmetric unit in space group *P*3_2_21. That solution had an initial correlation coefficient of 0.334 and an *R*_factor_ of 53.7%. Five percent of the reflections were excluded from the total for cross-validation with the *R*_free_. Initial structural refinements were carried out with CNS15) using diffraction data to 1.72 Å with several cycles of torsion-angle simulated annealing with an initial temperature of 2500 K, energy minimization, and individual *B*-factor refinement. After the structural refinements using CNS, structural refinements and auto model building were performed using ARP/wARP.16) After auto model building, several cycles of manual model rebuilding and model refinement were performed using XtalView17) and Refmac5.18) Water molecules were picked up from an *F*_o_-*F*_c_ map on the basis of peak heights and distance criteria. In the course of the water picking, four unexplained high electron density peaks were found. These were assigned as chloride ions and potassium ions considering the crystallization condition, the location where peaks observed, and the peak height of a 2*F*_o_-*F*_c_ and an *F*_o_-*F*_c_ map. Chloride was contained in the protein solution for crystallization as sodium chloride, and potassium was contained in the reservoir solution as sodium potassium tartrate. Evaluation of the quality of the model was performed with PROCHECK.19)

The coordinates have been deposited into the Protein Data Bank with the accession number 1V3Z.

## Results and discussion

The crystal structure of *P. horikoshii* AcP was solved by molecular replacement at 1.72 Å resolution and refined to an *R*_factor_ of 16.9% and an *R*_free_ of 19.0% with a good geometry. The asymmetric unit contained two molecules of *P. horikoshii* AcP. The final electron density allowed positioning of 90 residues in each molecule. We could not determine the position of the N-terminal methionine residue. The final model contained two chloride ions, two potassium ions, and 178 ordered water molecules. In the Ramachandran plot,20) 95.2% of the residues fell within the most favored regions, and the rest fell within the additionally allowed regions.

**Fig. 1:**
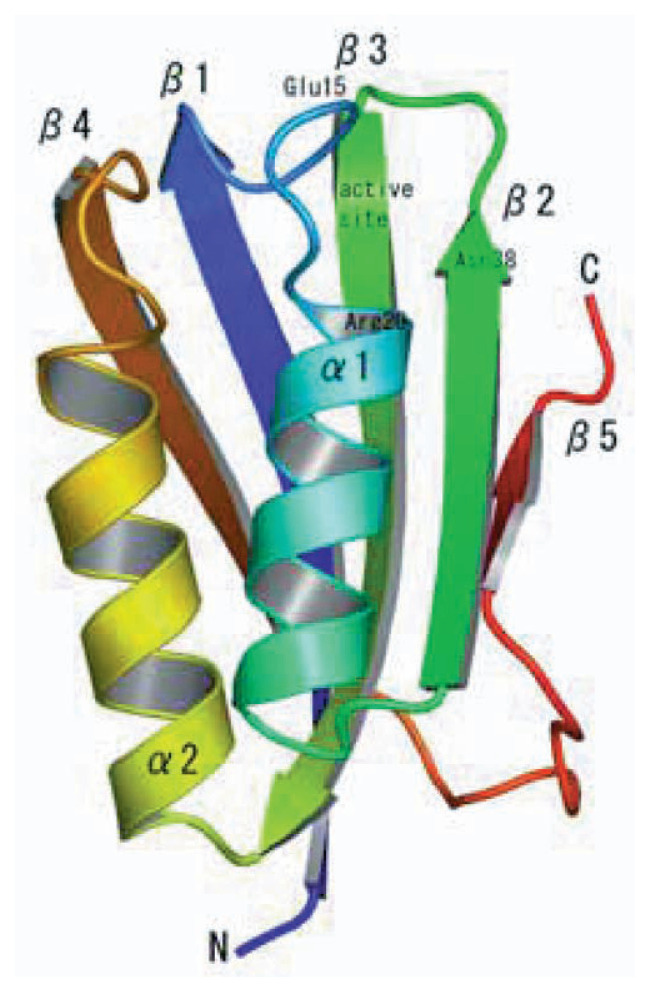
The overall structure of *P. horikoshii* AcP. Color coding runs from blue at the N-terminal region to red at the C-terminal region. Secondary structure assignments are labeled on the ribbon model.

*P. horikoshii* AcP displayed an ***α***/***β***-sandwich fold with a ***βαββαβ*** secondary structure composition (4-1-3-2-5 ***β***-strand topology; Fig. 1). The five-stranded ***β***-sheet was slightly twisted and faced one side of the antiparallel ***α***-helices. The dimensions of the *P. horikoshii* AcP molecule were about 33 Å × 23 Å × 20 Å.

The structure of *P. horikoshii* AcP was superposed with the known structures of other AcPs (bovine CT-AcP: PDB code 2ACY8) and AcPDro2: PDB code 1URR9)). This revealed that the structure of *P. horikoshii* AcP is very similar to those of other AcPs, except for a long loop positioned between ***β***4 and ***β***5. The root mean square deviation (r.m.s.d.) values calculated for the C*_α_* atoms were 0.66 Å (superposition of 84 residues of *P. horikoshii* AcP with bovine CT-AcP) and 0.88 Å (superposition of 82 residues of *P. horikoshii* AcP with AcPDro2).

**Fig. 2:**
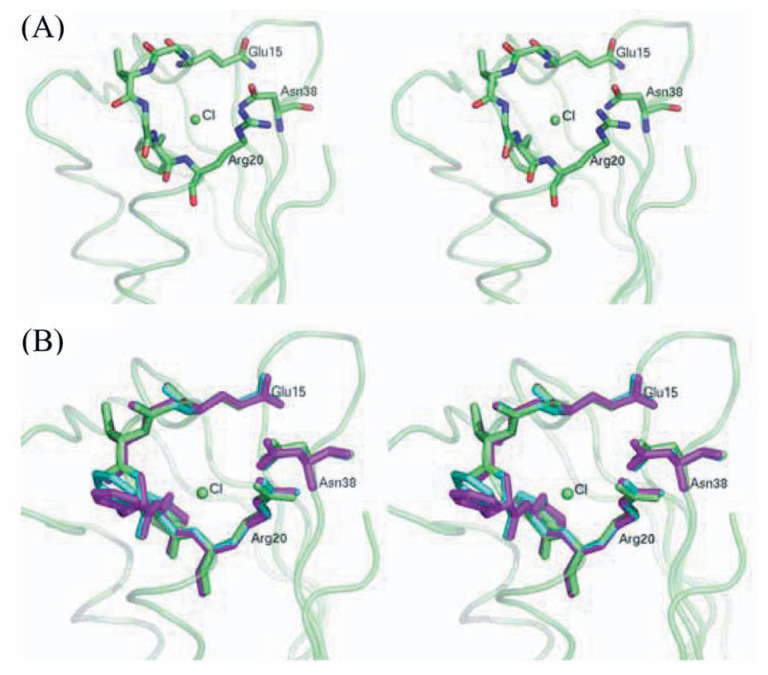
Stereo diagram of AcP active site. (A) The active site structure of *P. horikoshii* AcP. (B) Superposition of the AcP’s active site. Green, blue and pink stick models indicate the conformation of active site of *P. horikoshii* AcP, bovine CT-AcP and AcPDro2, respectively. The green sphere is the chloride ion in the *P. horikoshii* AcP structure.

Comparison of the structure and sequence of *P. horikoshii* AcP with those of eukaryotic AcPs allows us to identify the enzyme active site. The sequence stretch Gln15 - Arg20 forms a cradle-like conformation close to the N-terminal of the ***α***1 helix (Fig. 2(A)). In this region, the nitrogen atoms of the main chain point toward the center of the cradle, where the phosphate moiety of the substrate is expected to bind. Amino acid residues in this region are highly conserved among bovine CT-AcP, AcPDro2, and *P. horikoshii* AcP. This phosphate recognition mechanism is adapted by the low-molecular-weight phosphotyrosine protein phosphatases (LMW-PTPs).21)–23)

The critical residues for AcP enzyme activity have been suggested previously. In bovine CT-AcP, Arg23 and Asn41 are indispensable as the main phosphate binding residue and as the residue involved in the orientation and stabilization of catalytic water molecule, respectively. 8) These residues are conserved as Arg20 and Asn38 in *P. horikoshii* AcP. In the crystal structure of *P. horikoshii* AcP, we found chloride ion in the active site (Fig. 2(A)). Chloride ion is located at the center of the cradle-like pocket where sulfate and chloride ions, competitive inhibitors for AcPs, are positioned in the bovine CT-AcP structure. The chloride ion found in *P. horikoshii* AcP would also inhibit enzyme activity.

The conformation of active site was highly conserved among the CT-AcP, AcPDro2, and *P. horikoshii* AcP. The r.m.s.d. value calculated for the main chain atoms involved in the active site (Glu15 - Arg20 and Asn38) were 0.20 Å (superposition of 28 atoms of *P. horikoshii* AcP with bovine CT-AcP) and 0.27 Å (superposition of 28 atoms of *P. horikoshii* AcP with AcPDro2) (Fig. 2(B)).
